# Comparative dosimetric and radiobiological assessment of a hybrid-VMAT strategy incorporating supplemental short-arcs for left-sided postmastectomy radiotherapy

**DOI:** 10.3389/fonc.2026.1825312

**Published:** 2026-05-19

**Authors:** Xinyu Zhang, Yingtao Liao, Juan Shu, Ying Guo, Peixi Lan, Dingkui Zhang, Ze Chen, Fangfang Zhu, Xingyu Shen, Rikui Zhong, Fuchuan Xie

**Affiliations:** 1 Department of Radiation Oncology, HuiZhou Central People’s Hospital, Huizhou, China; 2 The First Clinical College of Hebei North University, Zhangjiakou, China; 3 Lanzhou Heavy-Ion Center, Gansu Wuwei Cancer Hospital, Lanzhou, China

**Keywords:** left-sided breast cancer, postmastectomy radiotherapy (PMRT), radiation dosimetry, radiobiological modeling, secondary cancer risk, volumetric modulated arc therapy (VMAT)

## Abstract

**Purpose:**

Postmastectomy radiotherapy (PMRT) for left-sided breast cancer poses persistent dosimetric challenges due to the complex target geometry and its proximity to critical organs at risk (OARs), particularly the heart and the left anterior descending (LAD) coronary artery. This study evaluated the dosimetric and radiobiological performance of a volumetric modulated arc therapy (VMAT) approach incorporating a supplemental short-arc segment, comparing it against conventional IMRT and standard VMAT strategies.

**Methods:**

Thirty-six left-sided breast cancer patients undergoing PMRT were retrospectively replanned using six techniques: 5-field and 7-field IMRT, tangential half-arc and wide-arc VMAT (THVP, TWVP), and two modified VMAT plans augmented with a 40° short-arc segment (sub-THVP, sub-TWVP) designed for coronary sparing. Plan quality was assessed via dose-volume histogram (DVH) metrics. Radiobiological evaluation included Normal Tissue Complication Probability (NTCP) via the Lyman-Kutcher-Burman and Poisson-LQ models, alongside secondary cancer risk (EAR/ERR) quantification using the full mechanistic Schneider OED model.

**Results:**

All VMAT-based techniques significantly improved target conformity and homogeneity over IMRT (p< 0.001). Among all evaluated strategies, sub-TWVP achieved the optimal trade-off between target coverage and cardiopulmonary sparing. It yielded the lowest ipsilateral lung exposure (V_5Gy_: 44.86%, V_20Gy_: 20.50%, D_mean_: 11.37Gy). Crucially, the short-arc integration in sub-TWVP significantly reduced cardiac exposure relative to standard TWVP, lowering heart V_5Gy_ and V_30Gy_ by 29.1% and 25.2% (p< 0.05), respectively, while achieving the lowest LAD D_mean_ (17.84 Gy). Radiobiologically, sub-TWVP yielded the lowest NTCP for the ipsilateral lung (0.045%) and minimized cardiac toxicity risks (0.16% for pericarditis, 0.36% for cardiac mortality). Furthermore, while standard VMAT inherently increased the low-dose bath to the contralateral lung compared to IMRT, the short-arc strategies subtly mitigated the corresponding modeled secondary cancer risk relative to their standard VMAT counterparts.

**Conclusion:**

Incorporating a 40° supplemental short-arc into tangential VMAT—particularly within a wide-arc configuration—significantly enhances cardiac and LAD sparing while preserving VMAT’s superior target coverage. Integrating complementary radiobiological modeling with DVH metrics provides critical insights for optimizing technique selection, effectively balancing normal-tissue toxicity against secondary cancer risks.

## 1 Introduction

Breast cancer is the most commonly diagnosed malignancy and a leading cause of cancer-related mortality in women worldwide (1). Approximately 2.3 million new cases are diagnosed annually, with roughly 670,000 deaths attributed to the disease (2). For patients presenting with high-risk pathological features or locally advanced disease, modified radical mastectomy followed by adjuvant postmastectomy radiotherapy (PMRT) constitutes a cornerstone of multidisciplinary management, significantly reducing locoregional recurrence and improving overall survival (3, 4). However, PMRT for left-sided breast cancer presents a persistent technical challenge. The target volumes, comprising the chest wall and regional nodes, exhibit complex, patient-specific geometries situated immediately adjacent to radiosensitive organs at risk (OARs), particularly the heart and the left anterior descending (LAD) coronary artery. The inclusion of regional nodal irradiation—most notably the internal mammary nodes (IMNs)—further expands the target volume. This often necessitates broader beam arrangements, intrinsically increasing the risk of acute and late normal-tissue toxicities (5). Therefore, highly conformal radiotherapy techniques are imperative to maintain robust target coverage while minimizing radiation exposure to surrounding healthy tissues.

To navigate these anatomical constraints, clinical practice has transitioned from conventional 3D-CRT to intensity-modulated radiotherapy (IMRT) and volumetric modulated arc therapy (VMAT). While IMRT improves target conformity (6, 7), its multi-field nature can increase delivery time and sensitivity to setup uncertainties (8). VMAT offers a more efficient alternative, utilizing continuous gantry rotation to achieve superior dose homogeneity while significantly reducing treatment times (9–11). Despite these advantages, a critical dosimetric trade-off persists in left-sided PMRT involving IMNs. To achieve robust IMNs coverage, conventional wide-angle VMAT inevitably scatters radiation across a larger volume of normal tissue. This expands the “low-dose bath”(e.g., V5) to the contralateral lung and breast, while simultaneously struggling to stringently restrict the mean dose to the LAD and the low-dose volume to the heart (12, 13). Importantly, many prior dosimetric studies have evaluated the chest wall and IMNs as a single amalgamated target (5). However, this simplified approach presents a significant limitation. The IMN chain is anatomically wedged between the chest wall and the cardiac anterior wall, necessitating exceptionally steep dose gradients to achieve coronary sparing. In contrast, the chest wall is a broader, more superficial volume. Amalgamating these structures into a single Planning Target Volume (PTV) forces the optimizer to apply generalized constraints across a massive region, which often masks localized high-dose spillage into the LAD. Therefore, separating these subvolumes is clinically and technically essential; it allows the treatment planning system to independently modulate the high-risk IMN-LAD interface and unmask the true sparing benefits of specific arc designs. This combined approach can obscure localized high-dose spillage and mask the specific dosimetric trade-offs occurring at the crucial LAD interface, highlighting the necessity of analyzing these subvolumes independently. As a result, questions remain regarding optimal technique selection, arc geometry design, and the delicate balance between cardiopulmonary sparing and the expanded low-dose bath in left-sided PMRT (14).

The clinical significance of this low-dose bath cannot be overstated, as expanded low-dose exposure correlates with an increased risk of both late cardiopulmonary toxicities and secondary malignancies. Particularly with the continuous improvement in breast cancer survivorship, the stochastic nature of radiation-induced carcinogenesis—modeled by the linear-no-threshold (LNT) paradigm—dictates that even minimal low-dose exposures carry cumulative, lifelong risks (15). Consequently, macroscopic dose-volume histogram (DVH) metrics alone are frequently insufficient to capture the full biological spectrum of these late-effect risks. To complement physical dosimetry, radiobiological indices—such as Normal Tissue Complication Probability (NTCP) and model-based secondary cancer risk metrics like Excess Absolute Risk (EAR) and Excess Relative Risk (ERR)—have been proposed (16–18). Importantly, treatment plans exhibiting similar macroscopic DVH characteristics can yield meaningfully different radiobiological predictions depending on how the dose is distributed spatially across an organ. Although a substantial body of literature has reported dosimetric comparisons for left-sided PMRT with regional nodal irradiation, a critical gap exists in the literature (3–5, 8, 9, 19, 20): few studies jointly evaluate advanced arc-optimization strategies alongside these radiobiological endpoints. This integrated evaluation is essential to accurately translate low-dose bath variations into quantifiable long-term survivorship risks.

In this study, we performed a comprehensive dosimetric and radiobiological comparison of IMRT and VMAT planning strategies for left-sided breast cancer with comprehensive IMNs irradiation. We implemented a systematic arc design strategy to minimize unnecessary low-dose exposure while preserving coverage of the chest wall and IMNs subvolumes. Specifically, we proposed a novel optimization strategy—defined hereafter as ‘Hybrid-VMAT’—incorporating a supplemental 40° short-arc tailored for the IMN region. Our central hypothesis is that this targeted short-arc configuration provides the optimizer with increased degrees of freedom to superiorly “sculpt” the dose away from proximal coronary structures. Consequently, we hypothesized that this strategy would enable a simultaneous improvement in both physical cardiac dosimetry and predicted radiobiological outcome metrics (NTCP and secondary cancer risk) compared to standard VMAT and IMRT, without expanding the contralateral low-dose bath or compromising target coverage. To rigorously test this, dosimetric outcomes were assessed for the target volumes (PTV-CW and PTV-IMN) and key OARs alongside these complementary radiobiological endpoints. By translating physical dose distributions into quantifiable clinical impacts, our ultimate goal was to clarify technique-specific trade-offs and inform clinically feasible planning strategies that optimize the therapeutic ratio more effectively than standard techniques in left-sided PMRT.

## 2 Materials and methods

### 2.1 Patients

This retrospective planning study included 36 consecutive patients with pathologically confirmed left-sided breast cancer who underwent mastectomy and PMRT between January 2021 and December 2023. Inclusion criteria were: (1) pathologically confirmed left-sided breast cancer; (2) modified radical mastectomy; and (3) planned adjuvant radiotherapy with comprehensive regional nodal irradiation. Indications for IMN irradiation were determined by board-certified radiation oncologists according to institutional protocols, including one or more of the following: (i) ≥4 positive axillary lymph nodes; (ii) pathologically confirmed IMN involvement; (iii) tumors located in the medial or central breast quadrants with regional nodal metastasis; or (iv) 1–3 positive axillary lymph nodes with high-risk pathological features (e.g., grade 3 histology, lymphovascular invasion, HER2 positivity, or hormone receptor negativity). Patients with right-sided or bilateral breast cancer, or those with major cardiopulmonary dysfunction precluding standard radiotherapy were excluded. The study was approved by the Research Ethics Committee of Huizhou Central People’s Hospital. Patient demographics and disease characteristics are summarized in **Table 1**.

**Table 1:** Clinical characteristics of the 36 patients included in this study.

Number of trial patients	36
Age (Years)	
Median[range]	54.5 [44–70]
Mean ± SD	55.91 ± 8.251
T Stage (According to AJCC 8^th^ Edition) (%)	
T1	38.89%
T2	52.78%
T3	5.56%
T4	2.78%
N Stage (According to AJCC 8^th^ Edition) (%)	
N1	38.89%
N2	38.89%
N3	22.22%
Stage wise distribution (%)	
IIA	13.89%
IIB	19.44%
IIIA	41.67%
IIIB	2.78%
IIIC	22.22%
HER2neu receptor status (%)	
HER2neu positive (HR negative)	38.89%
HER2neu positive (HR positive)	25.00%
Luminal A type	16.67%
Luminal B type (HR negative)	19.44%
PTV-CW Volume [cm^3^]	
Median[range]	674.560 [439.67-1178.09]
Mean ± SD	712.433 ± 156.318
PTV-IMN Volume [cm^3^]	
Median[range]	6.358 [3.635-14.815]
Mean ± SD	6.999 ± 2.257

### 2.2 Target and OARs delineation

All patients were immobilized using a carbon-fiber breast board combined with a head-and-neck thermoplastic mask. They were positioned supine with both arms raised above the head, with the head gently rotated toward the contralateral side (~10°–15°). Computed tomography (CT) images were acquired on a CT simulator (Somatom Definition AS Open, Siemens Healthineers, Germany) with a 5 mm slice thickness in free-breathing mode, extending from the mastoid region to the inferior border of the L2 vertebral body. A 5 mm tissue-equivalent bolus was placed over the chest wall during CT simulation to ensure adequate dose build-up in the superficial tissues, and its position was marked on the skin. This bolus protocol was rigorously maintained throughout the entire course of radiotherapy.

The clinical target volume (CTV) encompassed the chest wall and regional nodal volumes, including the supraclavicular and infraclavicular regions. The internal mammary nodal region was contoured separately as CTV-IMN. PTVs were created by applying a 5 mm isotropic expansion to each CTV. Each PTV was then retracted 3 mm from the patient’s body surface to prevent excessive beam modulation in the build-up region by the treatment planning system. Adequate superficial target coverage was concurrently maintained via the physical bolus. Specifically, the PTV-IMN was defined by expanding the CTV-IMN by 5 mm in all directions except medially, strictly excluding lung tissue to prevent inadvertent pulmonary exposure. All target delineations were performed by the same physician in accordance with the Radiation Therapy Oncology Group (RTOG) atlas to minimize inter-observer variability (21).

OARs—including the ipsilateral lung, heart, LAD coronary artery, contralateral lung and breast, spinal cord, liver, stomach, thyroid, and humeral heads—were initially delineated using an AI-based auto-segmentation system (Datu Medical Co., Shanghai, China). Subsequently, all contours were comprehensively reviewed and manually edited by board-certified radiation oncologists. Given the 5 mm CT slice thickness and its potential for volume-averaging artifacts in small serial structures, the LAD was meticulously verified using appropriate cardiac window/level settings, tracing from the left main coronary artery bifurcation down to the cardiac apex.

### 2.3 Treatment planning

**Figure 1:**
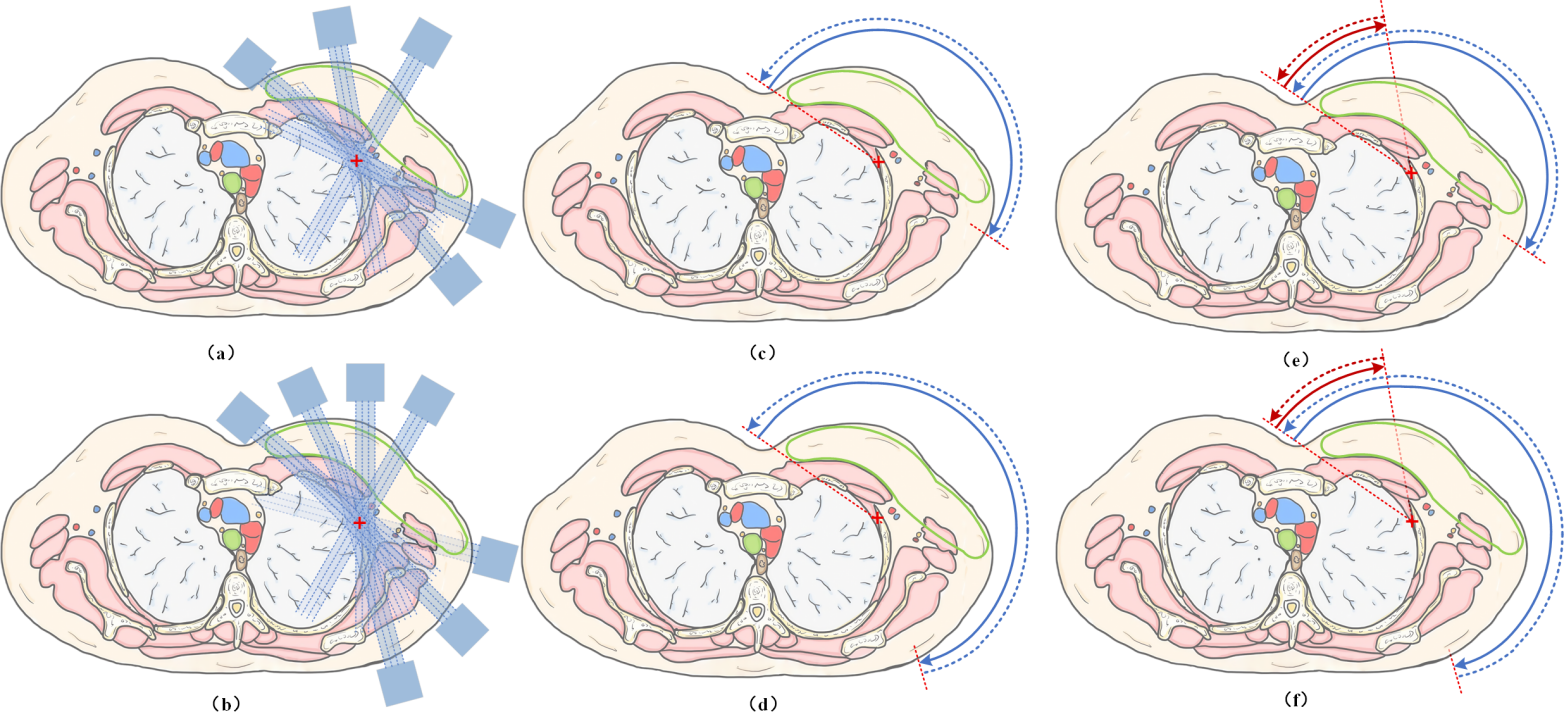
Field/arc arrangements for **(a)** 5F-IMRT, **(b)** 7F-IMRT, **(c)** THVP, **(d)** TWVP, **(e)** sub-THVP, and **(f)** sub-TWVP.

To address clinically relevant dosimetric trade-offs, six predefined treatment planning techniques were generated for each patient. Non-coplanar beam arrangements were intentionally excluded; preliminary evaluations indicated that for large, elongated targets like the chest wall and regional nodes, non-coplanar techniques offer limited dosimetric advantages while substantially increasing delivery complexity. **Table 2** summarizes the geometric parameters, and **Figure 1** illustrates the field/arc arrangements.

**Table 2:** Beam/arc geometry for the six planning techniques.

Techniques plan	Beam/arc arrangement (gantry angles)	Per angle increasement	Max control points per beam/Arc
5F-IMRT	300°, 340°, 20°, 105°, 125°	None	20
7F-IMRT	300°, 330°, 0°, 30°, 105°, 135°, 165°	None	20
THVP	Two partial arcs: 300°–120° (CW/CCW)	20°	160
TWVP	Two partial arcs: 300°–160° (CW/CCW)	20°	160
sub-THVP	THVP + short arc: 300°–340°	20°	160
sub-TWVP	TWVP + short arc: 300°–340°	20°	160

- IMRT Configurations: Two coplanar IMRT plans were created: a sparse 5-field IMRT (5F-IMRT) and a conventional 7-field IMRT (7F-IMRT). Baseline gantry angles were established to mimic standard clinical tangential trajectories. Patient-specific fine-tuning of these gantry angles was permitted exclusively within a narrow ±5° range to accommodate anatomical variations.
- Standard VMAT Configurations: Two VMAT plans were generated with arc start and stop angles explicitly matching the tangential orientations of the IMRT sets. The tangential half-arc VMAT plan (THVP) utilized two complementary partial arcs (clockwise and counterclockwise) spanning 180° (300°–120°) to emulate a conventional tangential approach. The tangential wide-arc VMAT plan (TWVP) extended the arc stop angle to 160° (a total span of 220°) to provide the optimizer with increased degrees of freedom, particularly for IMNs modulation. This 220° span upper limit was systematically determined via pilot testing, which revealed that further extensions disproportionately increased the contralateral low-dose bath without yielding clinically meaningful conformity gains.
- Hybrid-VMAT: Short-Arc Augmented Configurations (sub-THVP & sub-TWVP): To selectively spare the heart and LAD without extending the primary arc span, a supplemental short-arc segment was appended to the standard VMAT configurations. In this study, this Hybrid-VMAT strategy (Short-Arc Augmented VMAT) was implemented via two specific configurations: sub-THVP and sub-TWVP. The specific 40° span (300°–340°) was derived based on the geometric analysis of the LAD distribution and Beam’s Eye View (BEV) overlaps. Narrower spans (<40°) provided insufficient modulation capability for the IMNs, whereas wider spans increased the weighting of beams transiting the ipsilateral lung, incurring unacceptable pulmonary dose penalties.

### 2.4 Dose calculation and optimization

All plans were generated in Monaco^®^ TPS (Version 6.00.11, Elekta Solution AB, Stockholm, Sweden) for delivery on an Elekta Axesse^®^ linear accelerator (Elekta Oncology Systems, Crawley, UK) equipped with an Agility™ 160-leaf MLC using 6-MV photons. The system calculates radiation dose using the Monte Carlo Photon algorithm. A statistical uncertainty of 3% per control point and a 0.3cm dose calculation grid were utilized universally, reflecting a clinically validated balance between computational efficiency and dosimetric accuracy. The prescription was 50 Gy in 25 fractions to the chest wall and regional nodes. To establish a rigorous baseline benchmark, all plans were normalized according to ICRU Report 83 criteria, ensuring that at least 98% of the PTV received ≥95% of the prescription dose (V_47.5Gy_≥ 98%).

To ensure an unbiased comparison, all plans were generated by a single experienced medical physicist. Optimization objectives, priority rankings, and dose constraints were kept strictly identical across all techniques using a standardized template (**Table 3**). Regarding modulation constraints, up to 20 control points per beam were allowed for IMRT, whereas up to 160 control points per arc were permitted for VMAT. This variance reflects the inherent technical delivery mechanisms and standard clinical default settings of the respective modalities, rather than an optimization bias. Furthermore, all plans were deliverable with dynamic MLC at a nominal dose rate of 600 MU/min, and total monitor units were constrained below 1000 MU to maximize clinical delivery efficiency without prematurely restricting the optimizer.

**Table 3:** Planning parameters, weighting factors, and optimization goals for IMRT and VMAT in the Monaco TPS.

Structure	Cost function	Weight	Reference dose(Gy)	Power	Shrink margin(cm)	Isoconstraint	Goal
PTV-CW	Target Penalty	1				5000	V_50Gy_≥95%
	Underdose DVH	20	50			95	
	Quadratic Overdose	0.5	52			60	
PTV-IMN	Target Penalty	1				4850	V48.5Gy≥95%
	Underdose DVH	0.01	48.5			95	
	Quadratic Overdose	0.01	52			55	
Left Lung	Overdose DVH	150	5			45	V_5Gy_ ≤ 50%
	Overdose DVH	200	20			22	V_20Gy_ ≤ 25%
	Maximum Dose	0.1			0.3	5000	
Right Lung	Overdose DVH	15	5			5	V_5Gy_ ≤ 5%
Heart	Serial	10		1		800	D_mean_ ≤ 8Gy
	Overdose DVH	20	5			40	V_5Gy_ ≤ 45%
	Parallel	8	30	3.8		5	V_30Gy_ ≤ 5%
Right Breast	Serial	10		1		380	D_mean_ ≤ 4Gy
LAD	Overdose DVH	15	40			18	V40Gy ≤ 20%
	Serial	8		1		2200	D_mean_ ≤ 26Gy
Liver	Parallel	5	5	4		20	V_5Gy_ ≤ 20%
	Quadratic Overdose	0.01	5		1.2	2	
Stomach	Parallel	3	5	3.8		10	V_5Gy_ ≤ 15%
PRV Spinal Cord	Serial	0.1		6		2400	
	Maximum Dose	2				3200	Dmax ≤ 32Gy
Left Thyroid	Quadratic Overdose	0.01	38		0.5	80	
Right Thyroid	Quadratic Overdose	0.01	32			50	
Left Humerus Head	Parallel	5	30		3.8	20	V_30Gy_ ≤ 20%
Trachea	Quadratic Overdose	0.01	35		0.5	20	
Esophagus	Quadratic Overdose	0.01	38		0.5	20	
Brachial Plexus	Quadratic Overdose	0.01	40		0.5	50	
Skin	Quadratic Overdose	0.01	50		0.2	45	
	Quadratic Overdose	0.01	25		1.5	95	
	Maximum Dose	10				5450	
	Maximum Dose	0.5			0.3	5000	
	Maximum Dose	0.7			2.0	3500	
	Maximum Dose	1			5.0	2000	

### 2.5 Plan evaluation and radiobiological modeling

Dosimetric evaluation utilized standard DVH metrics. For the PTVs, D_2%_ (D_x_ represents the dose delivered to x% of the volume), D_98%_, D_50%_, and D_mean_ (mean dose to the PTV) were recorded. Specific target coverage metrics were explicitly assessed: V_47.5Gy_ (V_95%_, mandatory coverage), V_50Gy_ (V_100%_, prescription coverage), V_53.5Gy_ (V_107%_, standard hot-spot), and V_55Gy_ (V_110%_, strict hot-spot limit). Dose homogeneity was quantified using homogeneity index (HI) defined as HI = (D_2%_-D_98%_)/D_50%_ (22), where the ideal value is 0. Conformity was quantified using the Conformity Index CI= (V_PTV50Gy_/V_PTV_) × (V_PTV50Gy_/V_50Gy_) (23), where the ideal value is 1. In this equation, V_PTV_ represents the target volume, V_PTV50Gy_ denotes the PTV volume receiving ≥50 Gy, and V_50Gy_ is the total body volume receiving ≥50 Gy.

For OARs, D_max_, D_mean_ and relevant V_xGy_ metrics were evaluated for the ipsilateral lung, contralateral lung, heart, LAD, contralateral breast, and other contoured structures. Because contralateral organs predominantly receive low doses in IMRT/VMAT delivery, low-dose metrics (e.g., V_x_ and D_mean_) were explicitly included to characterize the “low-dose bath” while maintaining required target coverage. Additionally, radiation doses to the contralateral breast and lungs should be minimized as much as possible while maintaining adequate target coverage.

While HI and CI effectively reflect dose uniformity and spatial conformity, conventional DVH metrics lack comprehensive three-dimensional spatial distribution data. To better translate these physical dose disparities into predicted clinical outcomes, radiobiological endpoints were calculated based on the differential DVHs exported directly from the treatment planning system.

• Secondary cancer risk modeling (EAR/ERR): Model-based secondary cancer risk was estimated utilizing the Organ Equivalent Dose (OED) concept based on Schneider’s framework (24, 25). Rather than relying on a simple mean dose, the OED was calculated via voxel-wise integration of the differential DVH to accurately account for non-uniform dose distributions and critical radiobiological dynamics—specifically, cell sterilization, tissue repopulation, and proliferation. Because the evaluated OARs (lung and breast) are susceptible to carcinoma induction, the full mechanistic OED model for carcinomas was employed. Based on the total dose (*D*), dose per fraction (*d_F_*), and the repopulation (*R*) parameters, the OED was derived as follows:


$$\text{OED}=\frac{1}{V}\sum_{i}V_{i}\frac{e^{-{\text{α}}_{i}^{\prime }D_{i}}}{{\text{α}}_{i}^{\prime }R}\left(1-2R+R^{2}e^{{\text{α}}_{i}^{\prime }D_{i}}-{\left(1-R\right)}^{2}e^{-\frac{{\text{α}}_{i}^{\prime }R}{1-R}D_{i}}\right)$$

where *V_i_* is the fractional volume receiving dose *D_i_*. The dose-dependent cell killing parameter, $\alpha_{i}^{\prime }$, incorporates the linear-quadratic model parameters (*α* and *β*) and is adjusted for fractionation as follows:


$${\text{α}}_{i}^{\prime }=\text{α}+{\text{β}}_{LQ}D_{i}\frac{d_{F}}{D}$$

The Excess Absolute Risk (EAR, per 10,000 person-years) was subsequently computed based on the formulated OED utilizing the Preston-Schneider parametric method: $\text{EAR}=\text{OED}\cdot {\text{β}}_{\text{EAR}}\cdot \exp\left(\gamma_{e}\left(e-30\right)+\gamma_{a}\ln\left(a/70\right)\right)\cdot \left(1+\text{s}\right).$ Here, *β*_EAR_ denotes the initial slope of the dose-response model; *e* is the age at exposure; *a* represents the attained age; and *γ_e_* and *γ_a_* function as age correction factors. The parameter *s* accounts for gender-specific biological risk variations; given the exclusively female cohort, *s* was assigned +0.17. The specific parameter sets utilized were: lung (*β*_EAR_ = 7.5, *a* = 0.042, *R* = 0.83, *γ_e_* = 0.002 yr^-1^, *γ_a_* = 4.23 yr^-1^) and breast (*β*_EAR_ = 9.2, *a* = 0.044, *R* = 0.15, *γ_e_* = -0.037 yr^-1^, *γ_a_* = 1.70 yr^-1^), consistent with established literature (24, 25). The Excess Relative Risk (ERR) was obtained by normalizing the EAR to the baseline age- and sex-specific cancer incidence rate, *h*(*a*,*e*), in the reference population: *ERR* = *EAR*/*h*(*a*,*e*). Baseline incidence rates for female breast and lung cancers in the Chinese population were derived from the Global Cancer Observatory (GCO) CANCER TODAY database (26), specifically stratified by sex (female) and matched age cohorts.

• Normal tissue complication probability (NTCP): Biological indices serve as critical indicators for optimizing treatment trajectories by providing quantifiable estimates of expected OARs toxicity. To rigorously evaluate these risks, we employed two distinct radiobiological models tailored to specific clinical endpoints. The Lyman–Kutcher–Burman (LKB) model (27, 28), anchored in the concept of Equivalent Uniform Dose (EUD) (29), was utilized to assess parallel-tissue risks: radiation pneumonitis (grade ≥ 2) in the lung (TD_50_ = 30.5 Gy, m = 0.18, and n = 0.99) (30) and fibrosis in the breast (TD_50_ = 60 Gy, m = 0.12, n = 0.33) (31). Cardiac evaluation utilized the seriality (Poisson-LQ) model (32) to estimate the risk of radiation-induced pericarditis (TD_50_ = 49.2 Gy, γ = 3, s = 0.2) (33) and cardiac mortality (TD_50_ = 52.4 Gy, γ = 1.3, s = 1) (34), acknowledging the serial organization of critical cardiac substructures.

## 3 Results

### 3.1 Target coverage, conformity, and homogeneity

**Figure 2:**
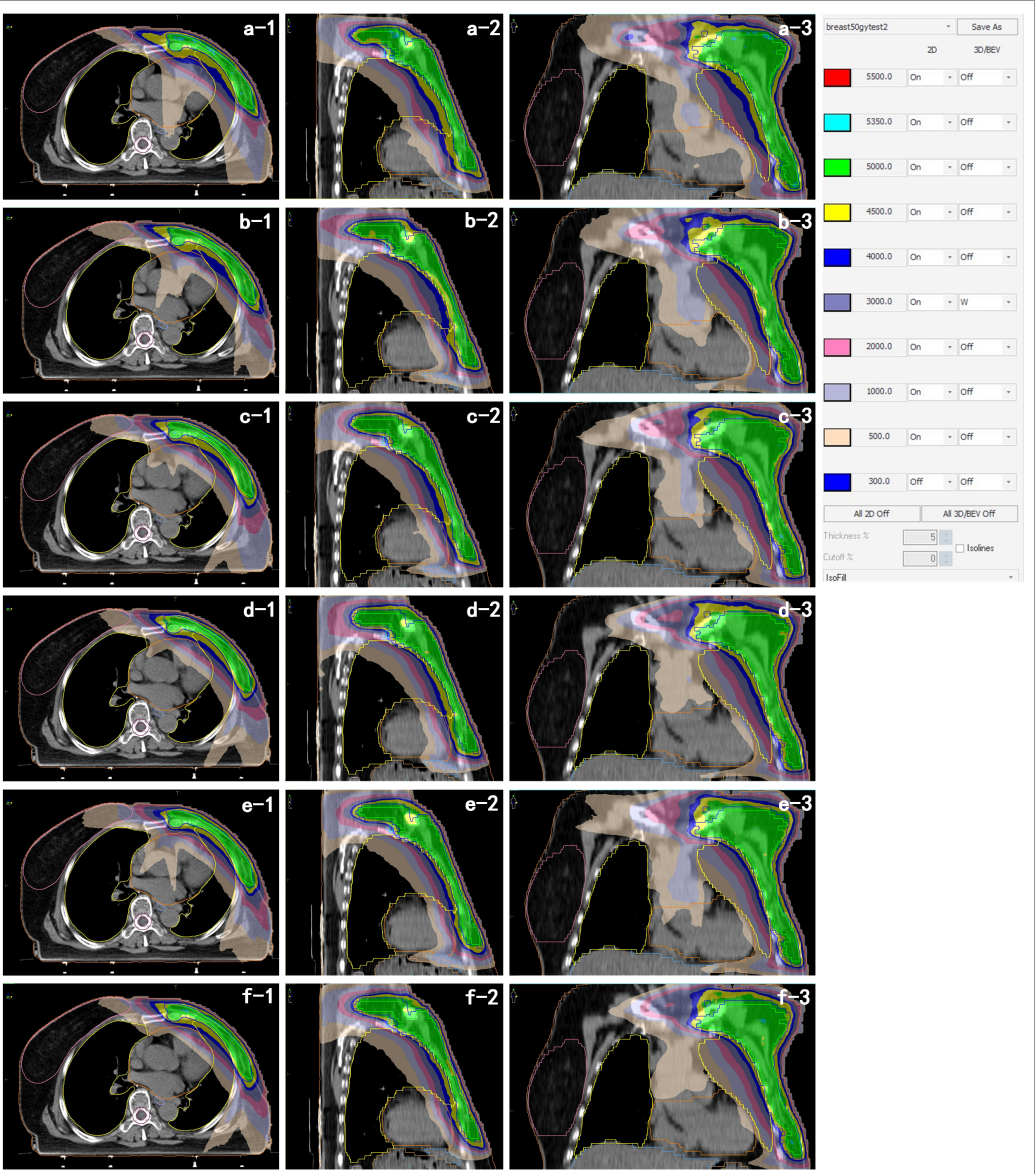
Representative dose distributions (axial, coronal, sagittal) for **(a)** 5F-IMRT, **(b)** 7F-IMRT, **(c)** THVP, **(d)** TWVP, **(e)** sub-THVP, and **(f)** sub-TWVP.

A total of 216 treatment plans (6 techniques × 36 patients) were generated and evaluated. Representative axial, coronal, and sagittal dose distributions are shown in **Figure 2**. All generated plans successfully met the mandatory clinical prescription criteria. Compared with IMRT, VMAT-based plans consistently produced lower high-dose spillage within the target and significantly improved both target conformity and homogeneity.

Comprehensive dosimetric indices for the PTV-CW and PTV-IMN are detailed in **Table 4**. All generated plans successfully met the clinical prescription criteria. Compared with IMRT plans, VMAT configurations achieved significantly higher V_47.5Gy_ for the PTV-CW (all p< 0.05) while simultaneously mitigating target hot-spots. Specifically, VMAT techniques substantially reduced the target hot-spot volume (V_53.5Gy_) compared to baseline IMRT. Within each VMAT pair (sub-TWVP vs. TWVP; sub-THVP vs. THVP), the addition of the short-arc segment did not yield statistically significant differences in target hot-spots (p > 0.05). For the PTV-IMN, all VMAT techniques similarly maintained excellent target coverage (V_47.5Gy_ > 99%) while controlling hot-spot volumes, confirming robust dose delivery to this challenging concave region.

**Table 4:** Comparison of PTV-CW and PTV-IMN of all IMRT and VMAT plans in this study.

Structures	Parameter	5F-IMRT	7F-IMRT	THVP	TWVP	Sub-THVP	Sub-TWVP	P-value
PTV-CW	D_98_% (Gy)							
	Median[range]	46.52 [43.41-47.68]	46.65 [45.57-47.69]	47.01 [46.19-48.24]	47.41 [46.43-48.22]	47.18 [46.12-48.08]	47.65 [46.42-49.10]	**a, **b, **c, *d, **e, **f, **g, *h, -i, **j, **k, **l, **m, *n, -o
	Mean ± SD	46.40 ± 0.913	46.71 ± 0.563	47.02 ± 0.480	47.42 ± 0.420	47.13 ± 0.514	47.63 ± 0.452	
	D_2_% (Gy)							
	Median[range]	53.61 [52.86-54.41]	53.48 [52.70-54.07]	53.24 [52.43-54.30]	52.91 [52.12-53.61]	53.11 [51.97-53.79]	52.86 [51.89-53.58]	**a, **b, **c, -d, **e, **f, **g, -h, *i, **j, **k, **l, **m, **n, *o
	Mean ± SD	53.64 ± 0.312	53.45 ± 0.351	53.22 ± 0.375	52.89 ± 0.337	53.08 ± 0.382	52.82 ± 0.397	
	D_50_% (Gy)							
	Median[range]	51.94 [51.28-52.91]	51.86 [51.07-52.50]	51.69 [50.76-52.27]	51.43 [50.68-52.13]	51.63 [50.73-52.32]	51.53 [50.09-51.98]	**a, **b, *c, *d, **e, **f, **g, -h, *i, **j, **k, **l, **m, *n, -o
	Mean ± SD	51.97 ± 0.304	51.83 ± 0.319	51.66 ± 0.323	51.43 ± 0.348	51.61 ± 0.346	51.43 ± 0.444	
	D_mean_(Gy)							
	Median[range]	51.57 [51.05-52.35]	51.53 [50.91-52.03]	51.36 [50.53-51.95]	51.18 [50.48-51.70]	51.31 [50.50-51.95]	51.32 [50.45-51.70]	**a, **b, -c, -d, -e, **f, *g, -h, -i, **j, **k, *l, **m, *n, -o
	Mean ± SD	51.59 ± 0.259	51.49 ± 0.281	51.34 ± 0.294	51.18 ± 0.277	51.31 ± 0.326	51.22 ± 0.324	
	V_47.5Gy_ (%)							
	Median[range]	97.01 [95.10-98.20]	97.14 [94.57-98.23]	97.26 [96.03-98.90]	97.85 [96.66-98.88]	97.57 [96.31-98.61]	97.77 [96.63-99.47]	**a, **b, **c, *d, *e, **f, **g, *h, -i, **j, **k, **l, *m, -n, -o
	Mean ± SD	96.95 ± 0.705	97.16 ± 0.689	97.35 ± 0.624	97.79 ± 0.579	97.53 ± 0.612	97.81 ± 0.633	
	V_50Gy_ (%)							
	Median[range]	89.10 [79.41-93.82]	89.21 [79.15-92.07]	90.18 [80.77-93.79]	91.61 [81.61-94.54]	90.34 [80.85-93.08]	91.73 [82.07-94.22]	-a, -b, -c, -d, -e, -f, -g, -h, -i, **j, *k, *l, *m, *n, -o
	Mean ± SD	88.976 ± 2.534	88.577 ± 2.900	90.594 ± 3.160	91.944 ± 2.819	90.908 ± 2.905	91.962 ± 2.458	
	V_53.5Gy_ (%)							
	Median[range]	2.67 [0.10-20.84]	1.86 [0.02-9.38]	1.18 [0.02-10.66]	0.42 [0-2.48]	0.45 [0-5.68]	0.26 [0-3.41]	**a, **b, **c, -d, -e, **f, **g, -h, -i, **j, **k, *l, **m, *n, -o
	Mean ± SD	3.92 ± 4.021	2.47 ± 2.304	1.67 ± 2.121	0.66 ± 0.690	0.91 ± 1.256	0.66 ± 0.923	
	V_55Gy_ (%)							
	Median[range]	0.01 [0-0.28]	0 [0-0.16]	0 [0-0.27]	0 [0-0.02]	0 [0-0.02]	0 [0-0.01]	*a, *b, -c, -d, -e, *f, *g, -h, -i, *j, *k, -l, -m, -n, -o
	Mean ± SD	0.024 ± 0.057	0.011 ± 0.028	0.011 ± 0.046	0.000 ± 0.003	0.001 ± 0.005	0.000 ± 0.001	
	HI							
	Median[range]	0.142 [0.114-0.210]	0.135 [0.104-0.169]	0.122 [0.098-0.155]	0.108 [0.081-0.143]	0.118 [0.093-0.147]	0.103 [0.072-0.143]	**a, **b, **c, -d, **e, **f, **g, -h, **i, **j, **k, **l, **m, **n, *o
	Mean ± SD	0.144 ± 0.210	0.135 ± 0.016	0.124 ± 0.013	0.109 ± 0.013	0.119 ± 0.013	0.104 ± 0.014	
	CI							
	Median[range]	0.77 [0.66-0.81]	0.78 [0.72-0.81]	0.8 [0.77-0.83]	0.81 [0.79-0.84]	0.8 [0.78-0.83]	0.82 [0.79-0.85]	**a, **b, **c, -d, **e, **f, **g, -h, **i, **j, **k, **l, **m, **n, -o
	Mean ± SD	0.764 ± 0.030	0.775 ± 0.021	0.797 ± 0.015	0.813 ± 0.013	0.799 ± 0.015	0.816 ± 0.014	
PTV-IMN	D_98_% (Gy)							
	Median[range]	47.12 [41.31-50.15]	47.85 [44.33-49.53]	48.14 [45.02-50.32]	48.66 [46.35-50.19]	48.56 [46.50-50.35]	48.46 [47.08-49.77]	**a, **b, **c, *d, -e, *f, **g, *h, -i, **j, **k, -l, **m, **n, -o
	Mean ± SD	47.31 ± 1.450	47.69 ± 1.096	48.02 ± 0.953	48.67 ± 0.719	48.62 ± 0.786	48.42 ± 0.634	
	D_2_% (Gy)							
	Median[range]	53.20 [52.30-54.46]	53.39 [51.97-54.76]	53.38 [52.16-54.15]	53.29 [52.22-54.64]	53.34 [52.18-54.08]	53.21 [52.48-54.27]	**a, *b, *c, *d, -e, *f, *g, *h, *i, -j, -k, -l, -m, -n, -o
	Mean ± SD	53.24 ± 0.506	53.328 ± 0.492	53.32 ± 0.529	53.35 ± 0.560	53.33 ± 0.461	53.32 ± 0.508	
	D_50_% (Gy)							
	Median[range]	51.50 [50.45-52.06]	51.56 [50.73-52.61]	51.78 [50.75-52.53]	51.76 [50.95-52.47]	51.74 [50.69-52.45]	51.95 [51.14-52.69]	**a, **b, *c, *d, -e, **f, **g, *h, -i, **j, **k, -l, **m, **n, -o
	Mean ± SD	51.39 ± 0.442	51.54 ± 0.414	51.73 ± 0.481	51.76 ± 0.397	51.73 ± 0.399	51.91 ± 0.417	
	D_mean_(Gy)							
	Median[range]	51.25 [50.04-51.87]	51.34 [50.50-52.27]	51.64 [50.73-52.42]	51.67 [50.61-52.31]	51.59 [50.51-52.35]	51.71 [50.89-52.43]	**a, **b, -c, *d, -e, **f, -g, **h, -i, **j, *k, -l, **m, *n, -o
	Mean ± SD	51.21 ± 0.451	51.31 ± 0.376	51.59 ± 0.462	51.58 ± 0.421	51.56 ± 0.404	51.69 ± 0.413	
	V_47.5Gy_ (%)							
	Median[range]	99.02 [84.03-100.0]	99.15 [93.09-100.0]	99.81 [94.42-100.0]	99.55 [95.71-99.96]	99.47 [96.31-100.0]	99.31 [97.14-100.0]	**a, **b, *c, *d, -e, **f, **g, *h, -i, **j, **k, -l, **m, **n, -o
	Mean ± SD	98.20 ± 2.771	98.28 ± 1.393	99.51 ± 0.976	99.34 ± 0.732	99.27 ± 0.795	99.09 ± 0.710	
	V_50Gy_ (%)							
	Median[range]	85.61 [61.77-98.66]	84.68 [74.08-95.44]	92.49 [73.11-98.93]	93.57 [76.78-98.41]	92.77 [73.73-98.63]	94.41 [80.56-99.27]	**a, **b, -c, -d, -e, **f, **g, -h, -i, **j, **k, -l, **m, **n, -o
	Mean ± SD	84.914 ± 8.701	84.951 ± 5.474	91.515 ± 6.518	90.878 ± 4.763	90.429 ± 5.495	90.563 ± 4.394	
	V_53.5Gy_ (%)							
	Median[range]	1.64 [0-15.77]	1.26 [0-13.32]	1.40 [0-13.01]	0.99 [0-13.2]	1.20 [0-8.21]	0.49 [0.01-9.25]	**a, *b, *c, *d, *e, -f, -g, *h, *i, -j, -k, *l, -m, -n, -o
	Mean ± SD	2.59 ± 4.127	2.07 ± 3.027	1.89 ± 3.0712	2.80 ± 3.766	2.25 ± 2.598	2.37 ± 2.799	
	V_55Gy_ (%)							
	Median[range]	0 [0-0.47]	0 [0-0.16]	0 [0-0.21]	0 [0-0.74]	0 [0-0.02]	0 [0-0.22]	-a, -b, -c, -d, *e, -f, -g, -h, -i, -j, -k, -l, -m, -n, -o
	Mean ± SD	0.019 ± 0.081	0.008 ± 0.031	0.011 ± 0.038	0.029 ± 0.127	0.001 ± 0.005	0.024 ± 0.058	
	HI							
	Median[range]	0.103 [0.057-0.239]	0.108 [0.073-0.186]	0.083 [0.056-0.147]	0.095 [0.060-0.117]	0.092 [0.060-1.052]	0.099 [0.071-0.122]	**a, *b, **c, **d, *e, *f, **g, **h, *i, *j, **k, -l, **m, **n, -o
	Mean ± SD	0.107 ± 0.032	0.113 ± 0.025	0.085 ± 0.020	0.096 ± 0.013	0.101 ± 0.167	0.098 ± 0.014	

a, sub-TWVP vs 5F-IMRT; b, sub-TWVP vs 7F-IMRT; c, sub-TWVP vs THVP; d, sub-TWVP vs TWVP; e, sub-TWVP vs sub-THVP; f, sub-THVP vs 5F-IMRT; g, sub-THVP vs 7F-IMRT; h, sub-THVP vs THVP; i, sub-THVP vs TWVP; j, TWVP vs 5F-IMRT; k, TWVP vs 7F-IMRT; l, TWVP vs THVP; m, THVP vs 5F-IMRT; n, THVP vs 7F-IMRT; o, 7F-IMRT vs 5F-IMRT; p indicates a statistical significance, **p ≤ 0.01, *p ≤ 0.05, -p>0.05.

Regarding overall plan quality, VMAT architectures consistently outperformed IMRT in terms of dose uniformity and spatial conformity for the chest wall. Wide-angle VMAT configurations (TWVP and sub-TWVP) yielded superior homogeneity (HI = 0.109 ± 0.013 and 0.104 ± 0.014, respectively) and higher spatial conformity (CI = 0.813 ± 0.013 and 0.816 ± 0.014, respectively) compared to both IMRT and half-arc VMAT configurations (p< 0.01). The integration of the supplemental short-arc modestly improved homogeneity while resulting in a slight reduction in conformity, reflecting a minor but clinically acceptable trade-off between dose uniformity and targeted spatial dose sculpting. Overall, sub-TWVP achieved the most favorable PTV metrics (lowest D_2_% and HI, highest D_98_% and CI), while stringently maintaining required target coverage.

### 3.2 OARs sparing analysis

**Figure 3:**
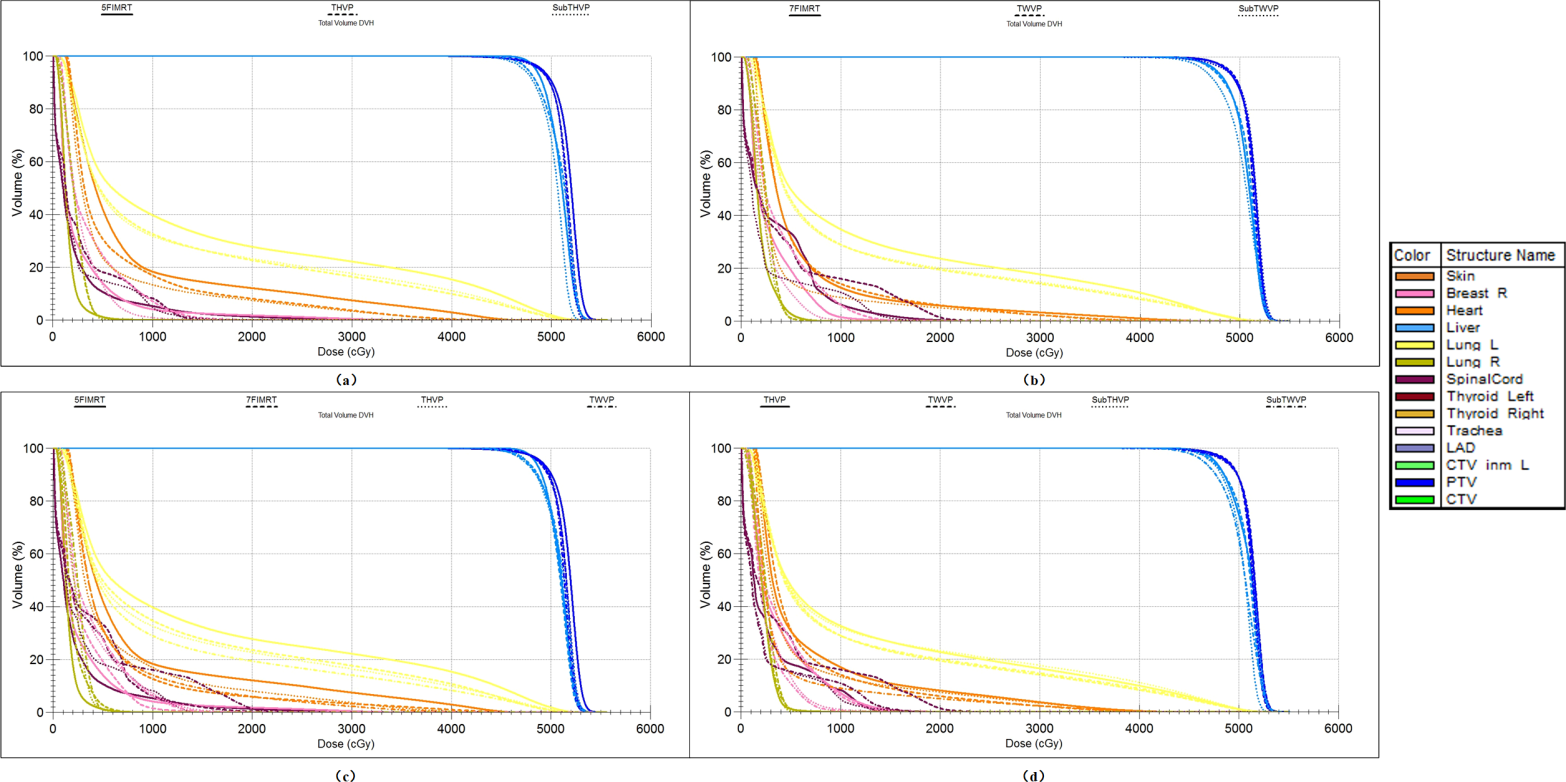
Group-averaged dose-volume histograms (DVHs) for the PTVs and OARs across selected treatment-plan comparisons (n = 36). **(a)** Comparison among 5F-IMRT, THVP, and sub-THVP; **(b)** comparison among 7F-IMRT, TWVP, and sub-TWVP; **(c)** comparison among 5F-IMRT, 7F-IMRT, THVP, and TWVP; and **(d)** comparison among THVP, TWVP, sub-THVP, and sub-TWVP.

Group-averaged DVHs for the PTVs and key OARs across selected treatment-plan comparisons are illustrated in **Figure 3**, and the corresponding dosimetric metrics are summarized in**Table 5**. Evaluation primarily focused on the ipsilateral lung, contralateral lung, heart, LAD, and contralateral breast.

**Table 5:** Dose comparison of OARs of all VMAT and IMRT plan in this study.

Structures/paramete	Data	5F-IMRT	7F-IMRT	THVP	TWVP	Sub-THVP	Sub-TWVP	P-value
ipsilateral lung								
V_5Gy_(%)	Median [range]	56.84 [39.63-65.60]	51.54 [39.44-60.04]	51.99 [39.34-60.93]	46.63 [34.32-51.19]	49.61 [37.92-57.78]	45.98 [33.19-48.92]	**a, **b, **c, *d, **e, **f, *g, *h, **i, **j, **k, **l, **m, *n, **o
	Mean ± SD	56.45 ± 4.849	51.12 ± 4.406	51.01 ± 4.773	45.72 ± 4.073	49.41 ± 4.572	44.86 ± 3.648	
V_10Gy_(%)	Median [range]	41.44 [27.70-48.61]	34.84 [26.12-42.73]	35.91 [27.16-42.44]	32.68 [22.15-35.76]	34.97 [26.16-41.74]	31.90 [22.74-35.09]	**a, **b, **c, *d, **e, **f, *g, *h, **i, **j, **k, **l, **m, *n, **o
	Mean ± SD	41.13 ± 3.870	35.18 ± 3.473	35.73 ± 3.818	31.66 ± 3.158	35.01 ± 3.671	30.85 ± 2.872	
V_20Gy_(%)	Median [range]	28.2 [20.13-36.44]	23.88 [18.2-30.76]	24.22 [18.96-30.96]	22.39 [14.17-24.86]	23.62 [18.68-29.53]	20.37 [14.83-24.62]	**a, **b, **c, *d, **e, **f, *g, *h, **i, **j, **k, **l, **m, *n, **o
	Mean ± SD	27.72 ± 3.059	24.45 ± 2.882	24.27 ± 3.041	21.29 ± 2.705	23.85 ± 3.000	20.50 ± 2.613	
V_30Gy_(%)	Median [range]	21.41 [15.29-29.76]	17.85 [11.91-23.58]	17.07 [12.55-22.12]	15.36 [9.81-18.05]	16.40 [12-21.62]	14.45 [10.42-17.97]	**a, **b, **c, *d, **e, **f, *g, *h, **i, **j, **k, **l, **m, *n, **o
	Mean ± SD	20.86 ± 3.040	17.55 ± 2.870	17.01 ± 2.702	14.80 ± 2.321	16.71 ± 2.620	14.45 ± 2.203	
D_mean_(Gy)	Median [range]	14.98 [11.51-18.28]	13.13 [10.53-15.88]	12.92 [10.24-15.24]	12.06 [8.80-14.27]	12.61 [10.03-14.87]	11.68 [9.04-12.96]	**a, **b, **c, *d, **e, **f, *g, *h, **i, **j, **k, **l, **m, *n, **o
	Mean ± SD	14.81 ± 1.405	13.09 ± 1.313	12.87 ± 1.402	11.69 ± 1.187	12.62 ± 1.260	11.37 ± 1.062	
Contralateral lung								
V_5Gy_(%)	Median [range]	1.43 [0.28-4.62]	2.27 [0.32-6.10]	2.96 [0.90-8.31]	2.84 [0.41-8.21]	2.78 [0.32-7.05]	2.55 [0.26-9.09]	*a, *b, *c, -d, *e, **f, *g, -h, -i, **j, **k, **l, **m, -n, **o
	Mean ± SD	1.97 ± 1.139	3.04 ± 1.334	3.55 ± 2.197	3.07 ± 1.660	2.96 ± 1.742	2.84 ± 1.758	
D_mean_(Gy)	Median [range]	1.66 [1.15-2.14]	1.97 [1.48-2.52]	2.50 [1.59-3.13]	2.53 [1.83-3.04]	2.47 [1.73-3.04]	2.41 [1.68-3.07]	**a, **b, *c, -d, *e, **f, **g, -h, -i, **j, **k, **l, **m, **n, **o
	Mean ± SD	1.64 ± 0.209	1.99 ± 0.265	2.50 ± 0.377	2.50 ± 0.258	2.44 ± 0.323	2.44 ± 0.305	
Heart								
V_5Gy_(%)	Median [range]	47.15 [23.99-57.52]	40.31 [11.42-56.1]	38.10 [20.03-57.28]	34.12 [19.27-54.05]	30.02 [7.40-51.31]	22.99 [7.15-43.94]	**a, **b, **c, **d, -e, **f, **g, **h, -i, **j, **k, *l, **m, -n, **o
	Mean ± SD	45.07 ± 8.586	38.11 ± 8.902	37.64 ± 9.613	33.61 ± 9.152	29.47 ± 10.070	23.84 ± 9.091	
V_30Gy_(%)	Median [range]	7.29 [0.29-12.55]	4.16 [0.08-10.32]	5.16 [0.41-9.4]	3.36 [0.08-8.22]	3.79 [0.06-7.67]	2.62 [0.03-5.84]	**a, **b, **c, **d, *e, **f, *g, *h, -i, **j, **k, *l, **m, -n, **o
	Mean ± SD	6.545 ± 3.393	4.138 ± 2.724	4.61 ± 2.430	3.37 ± 2.230	3.39 ± 1.929	2.52 ± 1.676	
D_mean_(Gy)	Median [range]	8.42 [4.45-10.54]	7.56 [3.16-9.69]	7.43 [3.992-10.34]	6.81 [3.92-8.65]	6.27 [2.69-8.17]	5.73 [2.51-7.63]	**a, **b, **c, **d, *e, **f, *g, **h, -i, **j, **k, *l, *m, -n, **o
	Mean ± SD	8.10 ± 1.550	7.02 ± 1.466	7.32 ± 1.589	6.59 ± 1.392	6.15 ± 1.424	5.43 ± 1.462	
LAD								
V_30Gy_(%)	Median [range]	46.65 [0.97-92.97]	35.16 [0.03-79.25]	39.96 [2.25-79.04]	30.75 [0-65.32]	31.93 [0.90-66.62]	21.03 [0-53.82]	**a, **b, **c, **d, *e, **f, -g, **h, -i, **j, **k, *l, -m, -n, **o
	Mean ± SD	45.35 ± 19.273	33.92 ± 14.855	38.26 ± 17.181	30.48 ± 14.924	31.50 ± 14.534	23.08 ± 12.974	
D_mean_(Gy)	Median [range]	25.41 [7.23-36.46]	22.31 [6.18-33.08]	23.35 [6.89-34.37]	21.09 [6.58-31.78]	20.62 [6.45-30.87]	18.24 [4.58-26.89]	**a, **b, **c, **d, *e, **f, -g, **h, -i, **j, **k, -l, -m, -n, **o
	Mean ± SD	24.98 ± 5.752	21.55 ± 5.110	22.59 ± 5.753	20.42 ± 5.362	20.53 ± 5.117	17.84 ± 4.953	
contralateral breast								
V_5Gy_(%)	Median [range]	12.27 [3.1-19.54]	5.98 [0.14-18.83]	22.06 [11.34-46.82]	10.35 [3.66-24.08]	22.09 [11.52-46.91]	10.41 [3.68-23.22]	**a, **b, **c, -d, -e, **f, **g, *h, **i, -j, **k, -l, -m, -n, **o
	Mean ± SD	12.48 ± 4.418	6.28 ± 3.657	21.82 ± 6.623	10.62 ± 4.262	21.91 ± 6.917	10.78 ± 4.168	
D_mean_(Gy)	Median [range]	3.01 [1.95-3.94]	2.48 [1.25-4.08]	3.77 [2.75-5.53]	2.89 [1.71-3.56]	3.84 [2.87-5.60]	2.92 [1.88-3.91]	**a, **b, **c, -d, -e, **f, **g, *h, **i, -j, **k, -l, -m, -n, **o
	Mean ± SD	2.99 ± 0.561	2.46 ± 0.640	3.78 ± 0.587	2.83 ± 0.482	3.89 ± 0.563	2.87 ± 0.486	
Left humeral head								
V_30Gy_(%)	Median [range]	17.15 [5.23-63.26]	14.56 [0.14-55.43]	4.88 [0-88.64]	7.60 [0-90.42]	4.61 [0-87.17]	4.59 [0-93.08]	*a, -b, -c, -d, -e, *f, -g, -h, -i, *j, *k, -l, *m, -n, *o
	Mean ± SD	20.57 ± 12.582	14.61 ± 9.656	11.11 ± 18.182	12.53 ± 18.716	11.47 ± 19.345	12.71 ± 21.213	
D_mean_(Gy)	Median [range]	20.02 [11.67-32.46]	18.91 [11.93-29.25]	19.31 [6.40-36.72]	19.60 [6.47-38.82]	18.55 [6.52-37.19]	19.29 [6.83-36.70]	*a, -b, -c, -d, -e, *f, -g, -h, -i, *j, *k, -l, *m, -n, -o
	Mean ± SD	20.41 ± 4.169	18.88 ± 2.841	19.58 ± 5.733	20.09 ± 6.005	19.46 ± 5.545	20.05 ± 5.603	
Left thyroid								
D_mean_(Gy)	Median [range]	45.11 [32.54-49.49]	46.40 [37.40-50.29]	46.44 [36.30-50.80]	46.42 [37.57-49.92]	46.98 [25.67-50.89]	46.66 [36.57-50.71]	*a, -b, -c, -d, -e, -f, -g, -h, -i, -j, *k, -l, -m, -n, *o
	Mean ± SD	44.27 ± 3.734	45.90 ± 2.794	45.74 ± 3.261	45.77 ± 3.053	45.93 ± 4.269	46.21 ± 3.101	
Right thyroid								
D_mean_(Gy)	Median [range]	23.24 [12.28-31.98]	19.48 [13.35-29.47]	19.42 [13.16-37.28]	21.87 [12.71-33.29]	23.42 [11.23-38.50]	24.76 [14.77-37.43]	*a, **b, **c, **d, *e, -f, *g, *h, -i, -j, **k, -l, -m, -n, **o
	Mean ± SD	23.46 ± 4.081	20.11 ± 3.740	20.03 ± 5.284	21.74 ± 4.360	23.05 ± 5.971	25.67 ± 5.035	

a, sub-TWVP vs 5F-IMRT; b, sub-TWVP vs 7F-IMRT; c, sub-TWVP vs THVP; d, sub-TWVP vs TWVP; e, sub-TWVP vs sub-THVP; f, sub-THVP vs 5F-IMRT; g, sub-THVP vs 7F-IMRT; h, sub-THVP vs THVP; i, sub-THVP vs TWVP; j, TWVP vs 5F-IMRT; k, TWVP vs 7F-IMRT; l, TWVP vs THVP; m, THVP vs 5F-IMRT; n, THVP vs 7F-IMRT; o, 7F-IMRT vs 5F-IMRT; p indicates a statistical significance, **p ≤ 0.01, *p ≤ 0.05, -p>0.05.

For the ipsilateral lung, both wide-angle techniques (TWVP and sub-TWVP) achieved the most favorable sparing, demonstrating significant reductions in low- to intermediate-dose volumes (V_5Gy_, V_10Gy_, V_20Gy_, V_30Gy_) and D_mean_ compared with both IMRT and half-arc VMAT configurations (p< 0.05). For contralateral structures, the sub-TWVP approach yielded the lowest contralateral lung low-dose exposure (V_5Gy_ = 2.84 ± 1.76%, D_mean_ = 2.44 ± 0.31Gy, p< 0.05). While these V_5Gy_ reductions appear numerically modest, minimizing this low-dose bath is clinically critical for mitigating late-onset radiation pneumonitis and long-term secondary malignancy risks.

Crucially, both short-arc augmented VMAT strategies substantially improved cardiac sparing relative to their standard VMAT counterparts. Specifically, sub-THVP achieved relative mean reductions in heart V_5Gy_, V_30Gy_, and D_mean_ of 21.70%, 26.46%, and 15.98%, respectively, when compared to standard THVP (all p< 0.05). Similarly, sub-TWVP reduced these cardiac metrics by 29.07%, 25.22%, and 17.60% relative to standard TWVP (all p< 0.05), comfortably satisfying the predefined clinical threshold of D_mean_ ≤ 8Gy. LAD exposure was significantly mitigated, with sub-TWVP yielding the lowest mean dose (17.84 ± 4.95Gy), consistent with the intended coronary sparing design. While expanding the primary arc span from 120° to 160° (TWVP vs. THVP) yielded modest improvements in cardiac sparing (p< 0.05), the most substantial and targeted dose reduction to the proximal coronary region specifically necessitated the incorporation of the predefined short-arc segment.

### 3.3 Radiobiological endpoint analysis

Model-based secondary cancer risk (EAR and ERR) estimates are summarized in **Table 6**, **7**. For the ipsilateral lung, VMAT-based techniques produced lower modeled risks than IMRT. Specifically, sub-TWVP yielded the lowest ipsilateral lung risk (EAR = 23.950 ± 14.404 per 10,000 person-years; ERR = 0.273 ± 0.102). Relative differences between short-arc augmented and corresponding standard VMAT plans were modest (sub-TWVP vs TWVP; sub-THVP vs THVP). Nevertheless, minimizing this metric remains a prudent objective for long-term breast cancer survivors. In contrast, for the contralateral lung, VMAT configurations increased the modeled secondary cancer risk compared with IMRT. This elevation is directly attributable to the larger low-dose exposure associated with continuous arc delivery, a phenomenon exacerbated by increased monitor units (MU), collimator leakage, and internal scatter. Among all techniques, standard THVP produced the highest contralateral lung risk. For the contralateral breast, wide-angle configurations (TWVP) generally reduced low-dose exposure compared to half-arc (THVP) designs by distributing the entrance and exit doses over a broader angular span. Importantly, the integration of the supplemental short-arc (sub-THVP and sub-TWVP) effectively mitigated the contralateral breast risk relative to their standard VMAT counterparts (e.g., EAR decreased from 7.941 in TWVP to 7.486 in sub-TWVP). This indicates that the targeted short-arc design not only successfully spares the cardiac structures but also aids the optimizer in controlling and reducing the low-dose scatter to the contralateral breast.

**Table 6:** Excess Absolute Risk (EAR) for patients obtained from Schneider.

Organ	EAR (Per 10,000 person years) [mean ± SD]					
	5F-IMRT	57F-IMRT	5THVP	5TWVP	Sub-THVP	Sub-TWVP
ipsilateral lung	28.513 ± 17.641	25.986 ± 15.683	25.972 ± 16.088	24.146 ± 14.792	25.873 ± 15.746	23.950 ± 14.404
contralateral lung	6.771 ± 4.086	8.750 ± 5.459	9.962 ± 6.203	9.658 ± 5.981	9.724 ± 6.085	9.386 ± 5.8627
contralateral breast	6.736 ± 1.333	5.887 ± 1.405	8.458 ± 1.342	7.941 ± 1.257	8.134 ± 1.442	7.486 ± 1.319

**Table 7:** Excess Relative Risk (ERR) for patients obtained from Schneider.

Organ	ERR [Mean ± SD]					
	5F-IMRT	7F-IMRT	THVP	TWVP	Sub-THVP	Sub-TWVP
ipsilateral lung	0.326 ± 0.123	0.299 ± 0.112	0.297 ± 0.108	0.277 ± 0.102	0.291 ± 0.104	0.273 ± 0.102
contralateral lung	0.077 ± 0.026	0.099 ± 0.034	0.114 ± 0.043	0.109 ± 0.044	0.113 ± 0.041	0.106 ± 0.039
contralateral breast	0.076 ± 0.030	0.068 ± 0.036	0.096 ± 0.039	0.090 ± 0.035	0.093 ± 0.37	0.086 ± 0.040

NTCP results for major OARs are summarized in **Table 8**. For the ipsilateral lung, wide-angle configurations (TWVP and sub-TWVP) yielded the lowest NTCP (0.0475 ± 0.0100% and 0.0447 ± 0.0085%, respectively), consistent with their reduced ipsilateral lung dose-volume exposure. Conversely, the NTCP of the contralateral lung for all VMAT plans was slightly higher than that of IMRT. Notably, absolute NTCP values for the contralateral lung and breast across all techniques were extremely low (<0.01%). At such microscopic levels, these model-predicted differences fall well below the clinical detection threshold. For cardiac endpoints (pericarditis and cardiac death), the short-arc augmented plans effectively mitigated risks. For instance, sub-THVP achieved a relative reduction in pericarditis NTCP of 24.7% and 16.4% for cardiac death compared with standard THVP. Similarly, sub-TWVP yielded relative reductions of 16.4% and 18.4% compared with standard TWVP. While baseline absolute cardiac NTCP values are already low in this cohort, these significant relative reductions quantitatively confirm the robust coronary-sparing efficacy of the short-arc design.

**Table 8:** Calculated normal tissue complication probability (NTCP) values in different techniques.

Organ	NTCP [Mean ± SD] (%)					
	5F-IMRT	7F-IMRT	THVP	TWVP	Sub-THVP	Sub-TWVP
ipsilateral lung	0.0818 ± 0.0183	0.0612 ± 0.0138	0.0592 ± 0.0141	0.0475 ± 0.0100	0.0563 ± 0.0124	0.0447 ± 0.0085
contralateral lung	0.0052 ± 0.0003	0.0057 ± 0.0004	0.0065 ± 0.0006	0.0065 ± 0.0004	0.0064 ± 0.0005	0.0064 ± 0.0005
Heart (pericarditis)	0.6567 ± 0.6901	0.3868 ± 0.5087	0.2417 ± 0.3100	0.1943 ± 0.2633	0.1821 ± 0.2862	0.1624 ± 0.2519
Heart (cardiac death)	0.9817 ± 0.6915	0.5934 ± 0.5180	0.4829 ± 0.3501	0.4371 ± 0.3118	0.4038 ± 0.3414	0.3569 ± 0.3215
contralateral breast	3.7301×10^-8^± 1.0226×10^-7^	1.2883×10^-7^± 2.7409×10^-7^	9.6398×10^-9^± 2.4234×10^-8^	3.7771×10^-9^± 4.2746×10^-9^	4.1104×10^-9^± 8.5492×10^-9^	3.5312×10^-9^± 5.3909×10^-9^

Overall, these findings establish EAR/ERR and NTCP models as indispensable tools for optimizing clinical decisions and rigorously evaluating spatial dose distributions that conventional DVH metrics alone fail to fully capture.

### 3.4 Monitor units and delivery time

Monitor units (MU) and estimated delivery time are summarized in **Table 9**. Compared with 5F-IMRT (690.27 ± 83.12 MU; 209.89 ± 23.09 s), THVP (854.53 ± 75.91 MU) required more MU (+23.81%) while substantially reducing treatment time to 109.89 ± 10.75 s (−47.64%). Similarly, TWVP (828.82 ± 79.19 MU) showed a modest MU increase of 4.60% relative to 7F-IMRT (792.33 ± 102.16 MU; 281.71 ± 45.01 s), while achieving a marked reduction in delivery time to 115.13 ± 11.90 s (−59.13%). Relative to the corresponding standard VMAT plans, the short-arc augmented strategies increased MU by 2.26% (873.87 ± 85.12 for sub-THVP vs. THVP) and 5.23% (872.11 ± 82.12 for sub-TWVP vs. TWVP), translating to moderate increases in delivery time to 135.60 ± 10.91 s and 142.93 ± 9.74 s, respectively (representing increases of 23.40% and 24.15%). Overall, VMAT improved delivery efficiency compared to IMRT, and the supplemental short-arc introduced only a moderate and clinically manageable increase in absolute delivery time.

**Table 9:** The Number of Monitor Units (MU) and treatment time with treatment plans. .

Parameter	5F-IMRT	7F-IMRT	THVP	TWVP	Sub-THVP	Sub-TWVP
MU[Mean ± SD]	690.27 ± 83.12	792.33 ± 102.16	854.53 ± 75.91	828.82 ± 79.19	873.87 ± 85.12	872.11 ± 82.12
Treatment time [Mean ± SD]	209.89 ± 23.09	281.71 ± 45.01	109.89 ± 10.75	115.13 ± 11.90	135.60 ± 10.91	142.93 ± 9.74

## 4 Discussion and conclusions

To the best of our knowledge, this is the first study to systematically integrate conventional DVH indices, normal tissue complication probability (NTCP), model-based secondary cancer risk (EAR/ERR), and delivery efficiency to evaluate a novel supplemental short-arc VMAT strategy against standard IMRT and VMAT techniques for left-sided PMRT. By jointly evaluating these multidimensional endpoints, we aimed to characterize technique-specific trade-offs that are often obscured when plan assessment relies on a single class of metrics.

The principal finding is that VMAT-based strategies significantly improved target conformity and homogeneity relative to standard 5-field and 7-field IMRT (p< 0.001), reflecting the inherent dosimetric advantages of continuous gantry modulation. More importantly, our proposed short-arc designs (sub-THVP and sub-TWVP) provided crucial additional cardiac and LAD sparing. Mechanistically, the 40° short-arc acts as a targeted modulation segment specifically aligned with the tangential IMN direction. This geometry effectively restricts anterior heart exposure and provides the optimizer with the necessary degrees of freedom to sculpt the high-dose gradients away from the proximal coronary vasculature. This enhanced cardiopulmonary sparing was achieved with highly acceptable impacts on overall plan quality; specifically, the conformity index experienced a marginal reduction of less than 0.01, and delivery time showed a manageable increase of approximately 24% relative to standard VMAT. Consequently, the sub-TWVP approach provides a highly favorable balance, combining robust target dose quality with minimized cardiac toxicity risks.

### 4.1 Rationale for baseline selection and technical evolution

Historically, conventional 3D-CRT has served as the benchmark for breast radiotherapy due to its inherent ability to minimize the “low-dose bath” (e.g., V_5Gy_) to contralateral tissues. However, for left-sided PMRT encompassing the IMNs, 3D-CRT’s ability to provide adequate target coverage while sparing adjacent cardiac structures is severely limited (35). To navigate these concave target geometries, 3D-CRT frequently forces substantially higher intermediate-to-high dose volumes (V_20Gy_ and V_30Gy_) to the ipsilateral lung and heart. This dosimetric compromise carries severe clinical consequences; population-based studies demonstrate that the incidence of grade ≥ 2 radiation pneumonitis is significantly higher in 3D-CRT cohorts compared to IMRT groups (23.1% vs. 6.4%) (36).

To mitigate these high-dose toxicities, clinical practice—particularly across major Chinese oncology centers—has largely transitioned to advanced delivery techniques such as IMRT and VMAT (37, 38). Current data indicate that IMRT yields a vastly superior balance of OAR sparing (especially concerning the lung V_20Gy_) and target precision by tailoring dose distributions to individual patient anatomies (7, 8, 39). While VMAT represents a further evolution toward delivery efficiency via continuous gantry rotation (9–11), both standard IMRT and VMAT still face limitations in sculpting steep dose gradients at the narrow IMN–coronary interface.

This specific technical gap justifies our focus on the Hybrid-VMAT strategy. By integrating a supplemental 40° short-arc, our method provides the optimizer with localized degrees of freedom that standard configurations lack. This allows for superior “dose sculpting” away from the LAD and heart without incurring the high-dose penalties of 3D-CRT or the excessive low-dose bath of unoptimized VMAT. Consequently, establishing IMRT and standard VMAT as benchmarks allows for the most rigorous and clinically relevant evaluation of the incremental benefits provided by our targeted arc-optimization approach.

### 4.2 Interpretation of target dose quality

All evaluated techniques strictly satisfied the target coverage requirement following a standardized normalization protocol (ensuring V_47.5Gy_ ≥ 98% across all plans) and identical maximum hotspot constraints in accordance with the ICRU Report 83 criteria. This rigorous baseline confirms that the observed dosimetric variations reflect genuine differences in physical dose shaping rather than inconsistent prescription scaling. Crucially, the technical necessity of separating the PTV-CW and PTV-IMN subvolumes addresses a primary shortcoming of modern arc-design optimization. In left-sided PMRT, the IMN chain is anatomically wedged immediately superficial to the cardiac anterior wall. Amalgamating these targets into a single PTV often forces the treatment planning system to apply generalized constraints across a massive region, which can easily ‘mask’ localized high-dose spillage at the narrow IMN–LAD interface. By utilizing an independent sub-volume analysis, we enabled the optimizer to apply granular, localized constraints, preventing macroscopic target metrics from obscuring critical dose-gradient conflicts in this high-risk region.

Analyzed within this independent sub-volume framework, VMAT plans demonstrated statistically superior conformity and homogeneity compared with IMRT. The CI of PTV-CW significantly improved from 0.764 ± 0.030 in baseline 5F-IMRT to 0.816 ± 0.014 in sub-TWVP, while the HI decreased from 0.144 ± 0.210 to 0.104 ± 0.014 (all p< 0.001). This consistent trend underscores the inherent dosimetric advantages of continuous gantry modulation and refined dynamic control over dose gradients (9, 19). Furthermore, wide-angle VMAT (TWVP/sub-TWVP) provided improved homogeneity and conformity over the half-arc designs (THVP/sub-THVP). The broader angular span (220° vs. 180°) increases the optimizer’s degrees of freedom to manage concave target geometries and regional nodal coverage, which is particularly relevant when IMNs are included. However, this geometric advantage is not without compromise. The extended beam paths traversing the patient inherently expand the low-dose bath to adjacent structures (e.g., increasing contralateral lung V_5Gy_) and marginally elevate monitor units, echoing the fundamental trade-off of arc-based delivery. Notably, appending the supplemental short-arc preferentially redistributes dose to reduce coronary-adjacent exposure. While this targeted modulation can slightly increase dose spillage outside the prescription isodose and marginally reduce the CI, this minor trade-off is highly acceptable in the clinical context of superior cardiopulmonary sparing.

### 4.3 Cardiopulmonary sparing and the value of short-arc supplementation

Left-sided PMRT is fundamentally constrained by the substantial anatomical overlap and proximity between the IMNs and the heart. A primary limitation of standard VMAT in this setting is that the continuous gantry rotation often necessitates beam trajectories that ‘sweep through’ the anterior cardiac volume to ensure robust IMNs coverage. Because current arc-optimization strategies often lack the localized degrees of freedom required to manage this high-gradient interface, they inevitably elevate heart V_5Gy_ and struggle to satisfy stringent LAD D_mean_ constraints without compromising target homogeneity. The 40° supplemental short-arc directly addresses these shortcomings. By providing targeted modulation that ‘sculpts’ the dose away from the proximal coronary region without sacrificing target coverage, the short-arc schemes achieved substantial reductions in cardiopulmonary exposure beyond the capabilities of conventional configurations. Specifically, the sub-TWVP approach reduced the mean heart dose (MHD) to 5.43 ± 1.46 Gy alongside parallel reductions in LAD dose. According to the large population-based study by Darby et al.—which estimates a 7.4% relative increase in the rate of major coronary events per 1 Gy increase in MHD (40)—this absolute dose reduction is clinically highly meaningful. This finding is consistent with recent evaluations of advanced arc therapy in complex concave target geometries (9, 15).

For the ipsilateral lung, wide-angle VMAT (TWVP/sub-TWVP) paradoxically demonstrated the most favorable low- and intermediate-dose metrics. This finding suggests that, when tangentially designed and appropriately optimized, the wide-angle geometry can reduce unnecessary pulmonary irradiation by effectively circumventing direct anterior-posterior entrance paths that would otherwise deeply traverse the ipsilateral lung tissue. Although the broader angular span inherently expands the contralateral low-dose bath, the sub-TWVP strategy remains highly advantageous. It serves as a focused “coronary-sparing add-on” that circumvents the need for complex, less robust non-coplanar solutions.

### 4.4 Low-dose bath and modeled secondary cancer risk

A key dilemma in modern breast RT is that techniques improving target conformity and heart sparing can also increase low-dose exposure to contralateral tissues. The EAR/ERR analysis clearly quantifies this trade-off: VMAT generally increased the modeled secondary cancer risk in the contralateral lung compared with IMRT, consistent with the broader distribution of low-dose radiation inherent to arc-based delivery (17, 18). For instance, standard TWVP increased the absolute EAR for the contralateral lung by approximately 2.89 per 10,000 person-years compared with 5F-IMRT (an ~42.6% relative increase, **Table 6**). Importantly, the short-arc augmented strategies successfully mitigated these risks relative to their standard VMAT counterparts. For example, sub-TWVP subtly reduced the contralateral lung EAR from 9.658 to 9.386, and significantly reduced the contralateral breast EAR from 7.941 to 7.486 compared to TWVP. While some absolute reductions are numerically modest, they indicate that arc design—specifically limiting irradiation paths directed at critical organs—can actively sculpt the low-dose exposure and influence its modeled consequences.

The clinical relevance of these modest modeled reductions must be interpreted through the lens of high long-term survivorship and the LNT model of radiation-induced carcinogenesis. Epidemiological data confirm the absence of an absolutely safe dose threshold for radiation-induced carcinogenesis. For instance, the ERR for secondary lung cancer increases by approximately 8.5% per Gy of mean lung dose (41), and mean exposures as low as 1 Gy can significantly elevate the risk to the contralateral breast (42). Against this clinical backdrop, our short-arc strategies stringently adhere to the ALARA (As Low As Reasonably Achievable) principle. By deliberately restricting contralateral irradiation, these optimization techniques translate subtle dosimetric gains into a meaningful mitigation of lifelong, dose-dependent stochastic risks.

This dosimetric-radiobiological complementarity is especially valuable when DVH differences appear trivial or contradictory. As a concrete example in our cohort, the sub-TWVP configuration yielded a slightly lower contralateral lung V_5Gy_ than 7F-IMRT (2.84% vs. 3.04%, respectively; **Table 5**); however, its modeled EAR was paradoxically higher (9.386 vs. 8.750; **Table 6**). This effectively captures the biological impact of differing spatial dose heterogeneities and internal scatter that V_5Gy_ alone obscures. By using the full mechanistic OED model (24, 25), we converted these spatial dose heterogeneities into quantifiable risk estimates. Such insights carry profound long-term implications, highlighting that minimizing the contralateral low-dose exposure must be a priority, particularly for younger patients.

Nevertheless, these results must be interpreted strictly as comparative decision-making tools rather than absolute clinical incidence predictions. EAR/ERR values are intrinsically dependent on parameter assumptions. Although we employed the full mechanistic OED model for carcinoma and utilized Chinese female-specific baseline incidence rates from the Global Cancer Observatory database, rigorous sensitivity analyses from the literature demonstrate that absolute risk predictions harbor substantial uncertainties. Furthermore, these physical dose-driven models do not account for confounding clinical factors, such as genetic susceptibility (e.g., BRCA mutations) or the carcinogenic impact of concurrent systemic therapies. Therefore, EAR/ERR metrics are best used to compare competing plans under consistent assumptions, guiding technique selection without over-promising absolute toxicity elimination.

### 4.5 NTCP findings and complementary plan assessment

The LKB-based and Poisson-LQ NTCP evaluations showed very low absolute predicted complication probabilities for several organs. However, even when absolute NTCP values are microscopically small (e.g.,<0.05%), the relative differences between techniques remain highly informative for optimizing plan safety. Interestingly, the predicted NTCP for cardiac death using the Poisson-LQ model was marginally higher than that for pericarditis. This discrepancy is directly attributable to the high relative seriality parameter (s=1) used for the cardiac death endpoint, acknowledging the serial organization of critical cardiac substructures (34). This makes the model highly sensitive to the localized high-dose regions (e.g., the LAD) typical in left-sided breast radiotherapy. Because the LAD governs ischemic mortality risk, this finding reinforces the strict clinical necessity of maintaining the LAD D_mean_ well below established thresholds. In contrast, pericarditis was modeled with a dominant parallel behavior (s=0.2), reflecting its dependence on global heart dose.

Crucially, the short-arc designs provided substantial, quantifiable reductions in cardiac risk. The sub-TWVP configuration achieved an approximate 16.4% relative reduction in pericarditis and 18.4% relative reduction in cardiac mortality compared to standard TWVP. These NTCP improvements strictly align with our observed reductions in heart and LAD DVH metrics. More broadly, this assessment underscores a fundamental limitation of conventional planning: competing techniques can appear similar on a subset of macroscopic DVH endpoints, yet diverge significantly in model-based risk estimates due to critical differences in the three-dimensional spatial distribution of the dose.

The radiobiological assessments in this study are grounded in organ-specific parameters derived from established breast cancer and long-term survivorship cohorts (24, 25, 27). However, the applicability of these models is subject to certain limitations. The primary uncertainty arises from model extrapolation, as the coefficients for secondary cancer risk (EAR/ERR) and tissue toxicity (NTCP) are derived from population-based data that may not fully capture individual biological variability or the synergistic effects of concurrent systemic therapies. Despite these inherent uncertainties, these mechanistic models provide a robust and standardized platform for the comparative evaluation of different arc designs, allowing us to translate physical dose gradients into quantifiable potential clinical risks.

### 4.6 Delivery efficiency and clinical feasibility

Beyond dose and modeled risk, deliverability is a practical determinant of technique adoption. VMAT substantially reduced estimated treatment time compared with IMRT, which minimizes intrafraction motion sensitivity and improves clinical throughput. Adding the short arc increased delivery time by ~24% relative to standard VMAT, but the total delivery time remained markedly lower than IMRT, and MU remained well within clinically manageable limits. Consequently, this modest increase does not impose excessive wear and tear on the linear accelerator, nor does it significantly elevate the risk of low-dose scatter-related side effects. To ensure seamless translation into routine practice, this strategy can be easily implemented using standardized class solutions with locked OAR constraint lists within the treatment planning system. It requires no supplementary therapist training for patient setup and remains fully compatible with standard patient-specific quality assurance (QA) protocols.

### 4.7 Limitations and future work

From a clinical translation perspective, we recommend considering the short-arc augmented wide-angle VMAT (sub-TWVP) as a highly viable routine practice for left-sided PMRT. However, several limitations inherent to this retrospective dosimetric study must be acknowledged.

First, the moderate cohort size derived from a single institution necessitates external validation to confidently assess generalizability. Second, LAD delineation can be inherently uncertain on non-contrast planning CTs. Because small inter-observer contouring variations—typically demonstrating spatial deviations of 2.4 to 4.4 mm (43)—can translate to substantial dosimetric discrepancies (e.g., variations of up to 10–15 Gy in D_max_ and coefficient of variations near 30% in D_mean_ (44)) in regions with steep dose gradients, future protocols should incorporate explicit contouring standardization and the use of planning risk volumes (PRVs). Third, radiobiological metrics are highly sensitive to modeling assumptions; they should be interpreted strictly as comparative decision-making aids rather than absolute incidence predictors. Finally, while this work evaluates planned dose distributions under free-breathing conditions, the Hybrid-VMAT strategy is intrinsically compatible with advanced motion management. For patients limited by pulmonary capacity or compliance, plan-level geometric optimization offers a vital safeguard for cardiac sparing (45), whereas DIBH-eligible patients may achieve synergistic benefits through ‘split-VMAT’ protocols (46). Despite the ~24% increase in delivery time (~143 s), the stability and sub-millimetric accuracy of multi-breath-hold delivery ensure clinical feasibility without compromising treatment robustness (47, 48). Future multi-institutional prospective studies incorporating delivery QA measurements, DIBH, and long-term survivorship follow-up are warranted to clarify the true clinical relevance of these modeled radiobiological risk differences.

### 4.8 Conclusions

In left-sided PMRT with IMNs irradiation, VMAT-based techniques provide improved target conformity and homogeneity compared with IMRT. Appending a supplemental short-arc segment to tangential VMAT further reduces heart and LAD exposure with acceptable trade-offs in conformity and deliverability. Among the evaluated strategies, the wide-angle short-arc approach (sub-TWVP) demonstrated the most favorable overall balance of target dose quality, cardiopulmonary sparing, and model-based radiobiological endpoints. These findings support the use of carefully designed arc geometries and complementary radiobiological modeling to inform technique selection and to optimize the therapeutic ratio in modern breast radiotherapy.

## Data Availability

The original contributions presented in the study are included in the article/supplementary material. Further inquiries can be directed to the corresponding authors.
